# Sustained Higher Levels of Plasma hsa-miR-17-5p Expression During Gestational Diabetes Mellitus and Postpartum

**DOI:** 10.3390/epigenomes9040037

**Published:** 2025-09-24

**Authors:** Arathi Pillai, Sibin M Kandi, Nidhi Tripathy, Deeptika Agarwal, Indrani Mukhopadhyay, Bhasker Mukherjee, Y Vashum

**Affiliations:** 1 Department of Biochemistry, Armed Forces Medical College, Pune 411040, India; arathipillai98@gmail.com (A.P.); deeptika82@gmail.com (D.A.);; 2 Multi Disciplinary Research Unit, Armed Forces Medical College, Pune 411040, India; nidhimtripathy@gmail.com; 3 Department of Obstetrics & Gynecology, Armed Forces Medical College, Pune 411040, India

**Keywords:** gestational diabetes mellitus, miR-16, miR-17, miR-20, biomarker, postpartum

## Abstract

**Background:** The role of circulatory miRNAs in gestational diabetes mellitus (GDM) was explored extensively in previous studies. However, there was limited literature on longitudinal studies exploring the changes in miRNA expression during pregnancy and postpartum to understand the changes in their expression levels in GDM patients. **Methods:** Blood samples from thirty GDM subjects and twenty normoglycemic pregnant women (NGT) were collected between 24 and 28 weeks of their pregnancy, and follow-up samples from the same subjects were collected till 12 weeks postpartum (FGDM and FNGT, respectively). Three candidate miRNAs, hsa-miR-16-5p, hsa-miR-17-5p, and hsa-miR-20a-5p, were quantified from their plasma samples using RT-qPCR. Comparative analysis of these miRNA expression levels was made between different groups. **Results:** hsa-miR-16-5p, hsa-miR-17-5p, and hsa-miR-20a-5p expression were significantly higher in GDM patients when compared to NGT subjects. Interestingly, hsa-miR-17-5p has shown consistent upregulation in FGDM even after these patients turned normoglycemic. Additionally, hsa-miR-16-5p was found to be higher in FGDM patients compared to FNGT subjects. **Conclusions:** The present study corroborated the finding of differential expression of hsa-miR-16-5p, hsa-miR-17-5p, and hsa-miR-20a-5p in GDM. It also marked the importance of monitoring the levels of hsa-miR-17-5p and hsa-miR-16-5p during pregnancy and postpartum in GDM patients.

## 1. Introduction

Gestational diabetes mellitus (GDM), a prevalent metabolic disorder of pregnancy, is defined as glucose intolerance with onset or first recognition during gestation [1,2]. Globally, GDM affects approximately 7–10% of pregnancies, a rate that continues to rise in parallel with increasing obesity and maternal age [3]. It is associated with multiple adverse outcomes, including pre-eclampsia, fetal overgrowth, neonatal hypoglycaemia, and long-term risks of type 2 diabetes mellitus (T2DM) for both mother and offspring [4,5]. The conventional diagnosis, based on the oral glucose tolerance test (OGTT) performed during the second trimester, often captures the disease after metabolic alterations have already impacted placental and fetal development [6,7]. GDM women’s glucose levels go back to normal postpartum in the majority of cases; however, a smaller fraction of them remain hyperglycaemic [8,9,10,11]. As the majority of patients refuse to undergo OGTT postpartum, a fasting blood glucose test is often used for determining hyperglycemia state and ruling out prediabetes or T2DM. Hence, a robust biomarker that is least affected by confounding variables and more stable is warranted for postpartum diagnosis of T2DM.

MicroRNAs (miRNAs), small (~22 nucleotide) non-coding RNAs, have emerged as a promising non-invasive marker for early detection of GDM due to their stability in bodily fluids, resistance to degradation, and regulatory influence over multiple biological pathways, including glucose metabolism, insulin signalling, and placental development [12,13,14,15,16]. Moreover, meta-analyses consolidating data across diverse populations have highlighted a set of consistently dysregulated miRNAs, despite variations in study design, ethnicity, and sample type [17]. This growing body of evidence not only underscores the potential of miRNAs as predictive markers but also highlights the necessity for standardized methodologies in miRNA profiling to ensure reproducibility and clinical translation. Specifically, upregulation of miR-29a-3p, miR-16-5p, miR-17-5p, miR-20a-5p, and miR-330-3p, and downregulation of miR-132 and miR-155 have been consistently observed across the studies [18]. These miRNAs were found to be associated with the severity of maternal and fetal outcomes in GDM, suggesting their dual utility as both a diagnostic and prognostic biomarker [19].

However, there are a limited number of studies that have quantified the levels of miRNAs during GDM and postpartum to understand the changes in miRNA expression during postpartum normoglycemia [14,18]. The question remains whether the dysregulated miRNAs will come back to normal levels during normoglycemia in the postpartum stage. Hence, the present study evaluated the changes in the levels of three shortlisted candidate miRNAs (hsa-miR-16-5p, hsa-miR-17-5p, and hsa-miR-20a-5p) between 24 and 28 weeks of gestation and till 12 weeks postpartum in GDM subjects and healthy pregnant women.

## 2. Results

### 2.1. Clinical Characteristics and miRNA Expression Levels in NGT vs. GDM

**Figure 1:**
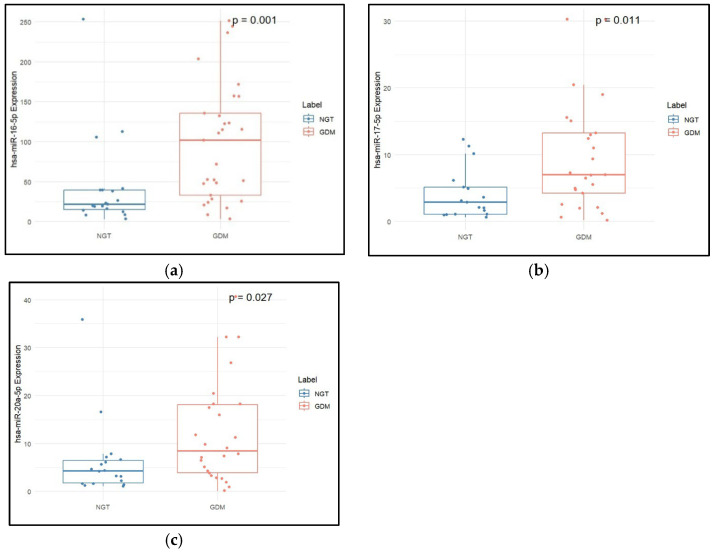
Expression levels of hsa-miR-16-5p (**a**), hsa-miR-17-5p (**b**), and hsa-miR-20a-5p (**c**) in pregnant individuals with GDM and normoglycemia (NGT). The median and interquartile range values are given in the box, and the highest and lowest values are given in the whiskers. As the data was not distributed normally, the two-sample Wilcoxon test was used for comparison of data in two groups.

**Figure 2:**
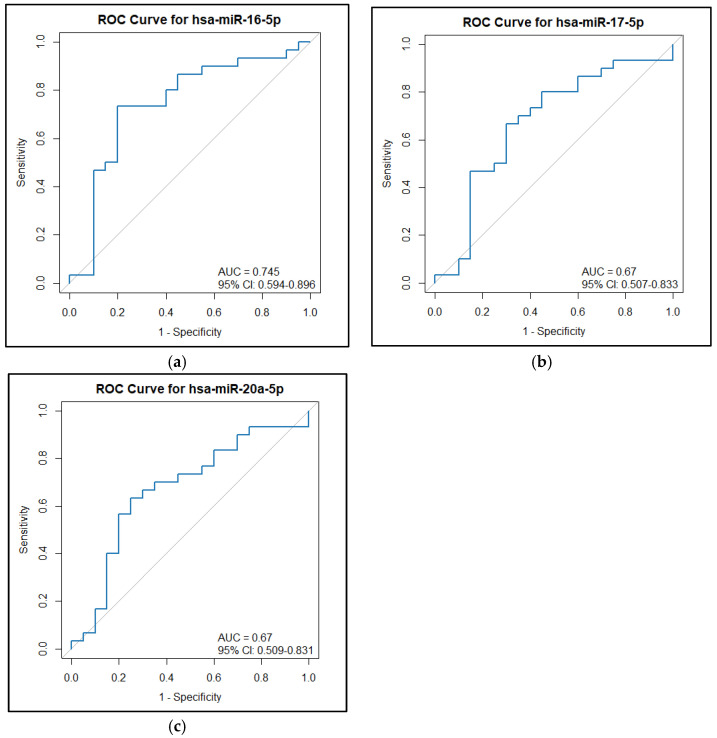
ROC curve analysis of (**a**) hsamiR-16-5p, (**b**) hsa–miR-17–5p, and (**c**) hsa–miR–20a–5p expression in the NGT group and GDM group.

BMI, SBP, and TG were found to be significantly higher in the GDM group as compared to the NGT group. However, LDL-c was significantly lower in the GDM group as compared to the NGT group. All three miRNAs, hsa-miR-16-5p (*p* = 0.004), hsa-miR-17-5p (*p* = 0.044), and hsa-miR-20a-5p (*p* = 0.044), were found to be significantly overexpressed in the GDM group compared to the NGT group, as shown in Table 1 and Figure 1. The diagnostic utility of these miRNAs was assessed using an ROC curve analysis. In ROC curve analysis, hsa-miR-16-5p showed an AUC of 0.745 and a sensitivity of 0.733 (0.833–1) at a specificity of 0.8 (0.4–0.95) with a cut-off of 44.38. hsa-miR-17-5p showed an AUC of 0.67 and a sensitivity of 0.667 (0.7–1) at a specificity of 0.7 (0.4–0.9) with a cut-off of 6.312. hsa-miR-20a-5p showed an AUC of 0.67 and a sensitivity of 0.633 (0.7–1) at a specificity of 0.75 (0.35–0.95) with a cut-off of 7.299 (Figure 2).

### 2.2. Clinical Characteristics and miRNA Expression Levels in GDM vs. FGDM

**Figure 3:**
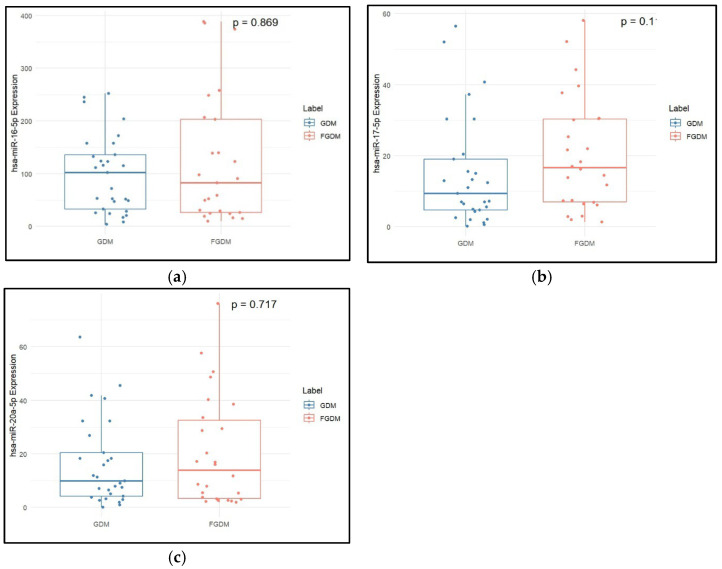
Expression levels of (**a**) hsa-miR-16-5p, (**b**) hsa-miR-17-5p, and (**c**) hsa-miR-20a-5p in post-delivery individuals with GDM and their corresponding samples during pregnancy. The median and interquartile range values are given in the box, and the highest and lowest values are given in the whiskers. As the data was not distributed normally, the two-sample Wilcoxon test was used for comparison of data in two groups.

Serum TGs and HDL-c were found to be significantly higher in the FGDM when compared to the GDM group, as shown in Table 2. Expression of hsa-miR-17-5p was significantly increased in the FGDM group when compared to the GDM group (*p* value = 0.026). Figure 3 is a box plot showing the relative expression of miRNAs in GDM and FGDM groups.

### 2.3. Clinical Characteristics and miRNA Expression Levels in FNGT vs. FGDM

**Figure 4:**
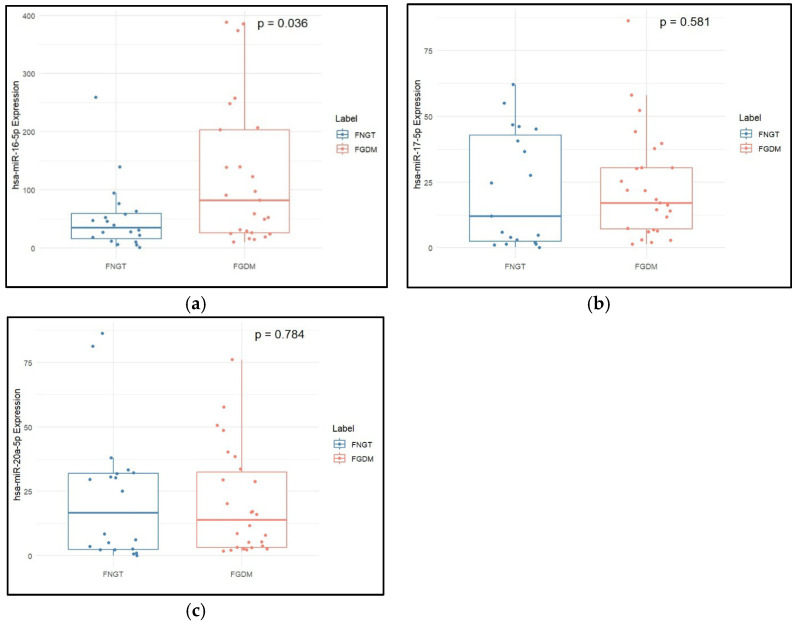
Expression levels of (**a**) hsa-miR-16-5p, (**b**) hsa-miR-17-5p, and (**c**) hsa-miR-20a-5p in post-delivery individuals with GDM and normoglycemia. The median and interquartile range values are given in the box, and the highest and lowest values are given in the whiskers. As the data was not distributed normally, the two-sample Wilcoxon test was used for comparison of data in two groups.

The postpartum fasting blood glucose was found to be significantly higher in the FGDM group (*p* value = 0.00078) than in the FNGT group. Expression of hsa-miR-16-5p was significantly higher in the FGDM group than in the FNGT group (*p* value = 0.005, Table 3). Figure 4 shows the boxplot of miRNA expressions in the follow-up groups of FGDM and FNGT.

### 2.4. Clinical Characteristics and miRNA Expression Levels in NGT vs. FNGT

**Figure 5:**
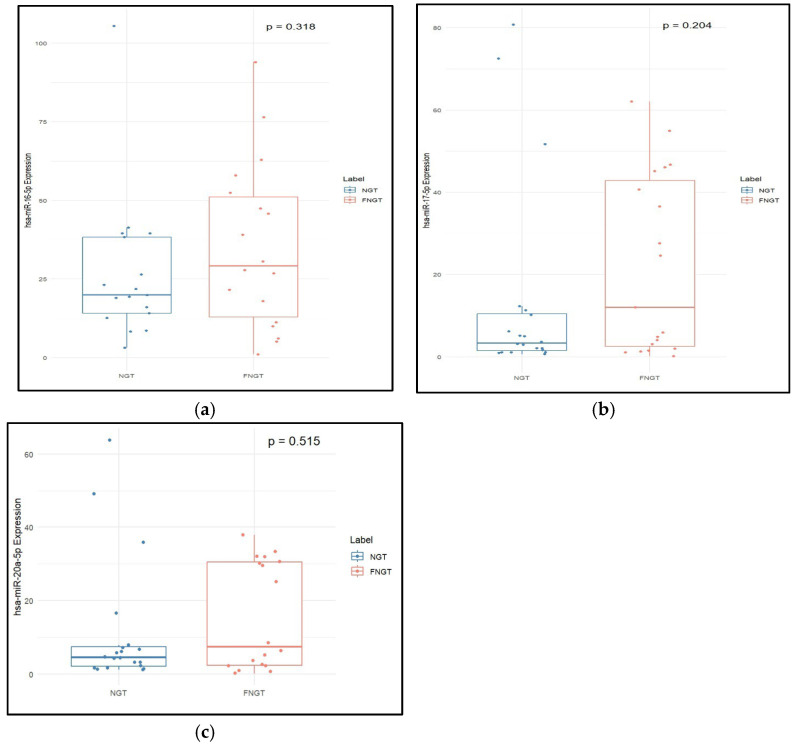
Expression levels of (**a**) hsa-miR-16-5p, (**b**) hsa-miR-17-5p, and (**c**) hsa-miR-20a-5p in post-delivery individuals with normoglycemia (FNGT) and their corresponding samples during pregnancy (NGT). The median and interquartile range values are given in the box, and the highest and lowest values are given in the whiskers. As the data was not distributed normally, the two-sample Wilcoxon test was used for comparison of data in two groups.

Total cholesterol and LDL-c were found to be significantly higher in NGT when compared to FNGT (Table 4). Expression of hsa-miR-16-5p, hsa-miR-17-5p, and hsa-miR-20a-5p was higher in FNGT compared to NGT, but these were not statistically significant (Figure 5).

### 2.5. Correlation Analysis

**Figure 6:**
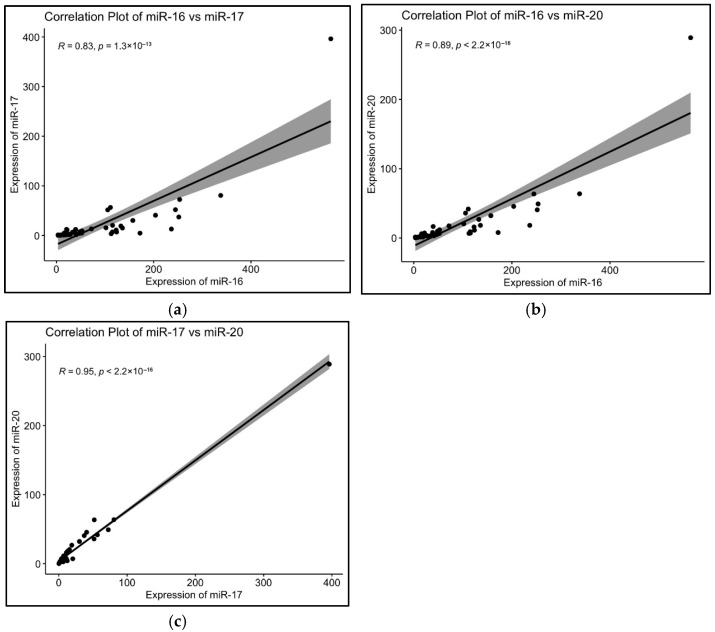
Correlation analysis of (**a**) hsa-miR-16-5p with hsa-miR-17-5p, (**b**) hsa-miR-16 with hsa-miR-20a-5p, and (**c**) hsa-miR-17-5p with hsa-miR-20a-5p.

hsa-miR-16-5p showed positive correlation with hsa-miR-17-5p with a rho value of 0.827 (*p* = 1.32 × 10^−13^) (Figure 6a). hsa-miR-16-5p also showed significant correlation with hsa-miR-20a-5p, with a rho value of 0.894, suggesting a positive correlation between the two (*p* ≤ 2.2 × 10^−16^) (Figure 6b). hsa-miR-17-5p and hsa-miR-20a-5p showed statistically significant correlation (*p* ≤ 2.2 × 10^−16^) with a rho value of 0.946, suggesting a positive correlation (Figure 6c). The complete results are given in Table S1.

## 3. Discussion

This study aimed to validate and extend previous research on the involvement of specific microRNAs in GDM. While hsa-miR-16-5p, hsa-miR-17-5p, and hsa-miR-20a-5p have been extensively studied and implicated in GDM previously, a key contribution of our work is the longitudinal analysis of their expression over the gestational period and postpartum. This allowed us to derive a link between GDM reversal, postpartum metabolic status, and candidate miRNA expression, a perspective often lacking in past studies that analyzed miRNAs at a single point in pregnancy [7,13,14,17].

In the present study, we observed a significant overexpression of hsa-miR-16-5p, hsa-miR-17-5p, and hsa-miR-20a-5p in women with GDM compared to normoglycemic controls (NGT). These miRNAs are known to be involved in key metabolic processes; hsa-miR-16-5p, part of the miR-15/16 family [20], is implicated in glucose metabolism and pancreatic beta-cell apoptosis [21,22]. Its overexpression may reflect an adaptive response to early glucotoxic stress in the maternal environment. Hsa-miR-17-5p and hsa-miR-20a-5p, belonging to the miR-17 family of miR-17-92 cluster [23], are known regulators of insulin signalling, cell proliferation, and lipid metabolism [24,25]. Their overexpression in our study may suggest dysregulation in metabolic pathways relevant to pregnancy-induced insulin resistance. Regarding their diagnostic utility, ROC curve analysis revealed that hsa-miR-16-5p, in particular, exhibited good diagnostic potential, suggesting its utility as a biomarker for GDM screening. This is consistent with other reports identifying miR-16-5p as a top classifier for identifying early-risk pregnancies with high accuracy. However, while the biological involvement of miR-17-5p and miR-20a-5p is suggested by their overexpression, ROC curve analyses for these two miRNAs showed modest AUC values (~0.67). These modest values indicate that, while hsa-miR-17-5p and hsa-miR-20a-5p may serve as exploratory signals for GDM, their individual diagnostic strength as standalone biomarkers is limited in our current findings. Therefore, their utility for GDM screening requires careful consideration, and further validation in larger, independent cohorts is essential to confirm their diagnostic potential, both individually and particularly when explored for enhanced predictive power in combined analysis with hsa-miR-16-5p. Furthermore, correlation analysis demonstrated strong positive associations among all three miRNAs. The high degree of co-expression indicates a shared regulatory mechanism, possibly through common transcriptional regulators or feedback loops within the same signalling pathways. These results are consistent with previous reports suggesting co-regulation of the miR-17-92 cluster in metabolic tissues [26].

Longitudinal follow-up revealed the persistent elevation of serum triglyceride levels, and HDL cholesterol levels increased significantly in the follow-up GDM (FGDM) group, aligning with known lipid abnormalities persisting postpartum in women with prior GDM [27]. hsa-miR-16-5p and hsa-miR-17-5p were significantly elevated in the follow-up GDM (FGDM) group postpartum, even after glucose regulation had reverted to NGT for some. Specifically, hsa-miR-16-5p levels were significantly higher in the FGDM group compared to FNGT, reinforcing its association with impaired glucose regulation beyond pregnancy and supporting its potential as a prognostic marker for glycemic deterioration [28]. However, this miRNA had not come back to a lower level in postpartum when compared to GDM. These findings support previous studies suggesting that miR-16-5p may serve not only as a diagnostic but also a prognostic marker for glycemic deterioration [28]. Multiple studies report elevated miR-16-5p in plasma, serum, urine-derived exosomes, and placentas from GDM pregnancies. It is detectable as early as the first trimester and remains high into the second and third trimesters [19]. miR-16-5p has been featured among top classifiers (with ~100% accuracy before validation, ~82% after cross-validation) for identifying early-risk pregnancies [29]. Elevated miR-16-5p correlates with HOMA-IR and high cardiovascular risk markers postpartum [19]. Placental urinary exosome studies suggest its expression contributes to insulin resistance and pro-inflammatory alteration during the second trimester [30]. A systematic review identified miR-16-5p (alongside miR-20a-5p, miR-222-3p, and miR-330-3p) as one of the most consistently dysregulated miRNAs in GDM, linked to β-cell proliferation, insulin secretion, resistance mechanisms, and cell-cycle regulation [17].

Similarly, hsa-miR-17-5p expression was significantly elevated postpartum in the FGDM group compared to baseline GDM, indicating its possible role in post-GDM metabolic transitions even in the absence of hyperglycemia. Elevated levels of plasma hsa-miR-17-5p at 16–19 weeks of gestation were observed in women who subsequently developed GDM, suggesting its potential as an early biomarker [31]. Another study analyzed peripheral blood samples from women 3–11 years postpartum who previously had GDM and found that four microRNAs—miR-17-5p, miR-16-5p, miR-29a-3p, and miR-195-5p—remained dysregulated in approximately 21% of these women. This suggests that specific miRNA signatures associated with GDM persist long term in maternal circulation. Such prolonged miRNA alterations may reflect ongoing metabolic or vascular stress, potentially contributing to an increased risk of developing type 2 diabetes and cardiovascular disease [32].

These findings align with previous studies showing persistent dysregulation of these miRNAs in women who are years postpartum and have a history of GDM, reflecting ongoing metabolic or vascular stress and potentially contributing to an increased risk of developing type 2 diabetes and cardiovascular disease [17,19,31,32]. Systematic reviews and longitudinal studies have found that plasma miR-17-5p is consistently elevated during all trimesters in women who develop GDM, correlating with insulin resistance. It is also associated with metabolic syndrome, cell proliferation, inflammation, mitochondrial dysfunction, and vascular damage. This suggests that circulating miR-17-5p elevation may reflect systemic metabolic stress [33,34]. Interestingly, the expression of these three miRNAs remained stable during delivery and postpartum, suggesting they are not significantly affected by peripartum molecular changes, making them suitable candidates for GDM diagnosis and monitoring.

Our study offers valuable insights into the longitudinal trajectories of hsa-miR-16-5p, hsa-miR-17-5p, and hsa-miR-20a-5p in GDM and postpartum, and benefits from a longitudinal design and the use of comparatively cheap and sensitive RT-qPCR technology. However, a notable limitation of our study is the relatively smaller sample size. Despite this limitation of sample size, the significant overexpression observed for hsa-miR-16-5p, hsa-miR-17-5p, and hsa-miR-20a-5p in women with GDM compared to normoglycemic controls and the strong diagnostic potential of hsa-miR-16-5p indicated by ROC curve analysis suggest a robust biological effect within our specific cohort. As this study was a longitudinal study, it was difficult to follow-up the patients till 12 weeks of postpartum samples were collected, thereby limiting the sample size. Nevertheless, this sample size, coupled with the study’s focus on a western Indian population, inherently limits the external validation and reproducibility of these findings in larger and more diverse epidemiological studies.

The consistent and strong associations detected, even in this cohort, underscore the potential clinical significance of these miRNAs but necessitate further exploration in larger, multi-ethnic cohorts to confirm generalizability and enhance predictive power, particularly for combined miRNA analyses. Furthermore, this study exclusively explored miRNAs; other epigenetic influences that might contribute to GDM pathology require further investigation. Our reliance on plasma samples rather than tissue samples also limits a comprehensive analysis of all molecular mechanisms of miRNA action on pancreatic cells, causing insulin resistance during pregnancy.

Another limitation of this study is the use of U6 snRNA as an endogenous control, despite ongoing debate regarding its stability across different sample types and experimental conditions [35,36,37]. While some studies report variability, others, including Silva et al. (2025) [38] and Duan et al. (2018) [39], have validated U6’s stability in specific contexts such as plasma and urinary sediment. In our study, U6 was chosen based on its historical use and inclusion in the cDNA kit, which also reduced additional costs. Although freeze–thaw cycles were controlled to ensure consistency, no initial validation of U6 stability was performed.

## 4. Materials and Methods

### 4.1. Sample Collection

The present study was conducted at the Department of Biochemistry, AFMC, Pune, from January 2023 to April 2025. Pregnant women reporting to the Outpatient Department (OPD) of the Obstetrics and Gynaecology department of the Southern Command Hospital, Pune, were recruited for the study. The study was approved by the institute’s ethical committee for human studies (IEC No. IEC/2022/08), and written informed consent was obtained from every subject before enrolment into the study. Thirty patients between 24 and 28 weeks of gestation who were diagnosed with gestational diabetes mellitus (ICD-10-CM O24) in the age range of 20–40 were included in the study (GDM, n = 30). These patients were followed up till 12 weeks postpartum, and blood samples were collected (FGDM, n = 30). The exclusion criteria for the study were hypertension, thyroid disease, obesity, malnutrition, any other chronic illness, genetic diseases, family history of diabetes mellitus, alcoholism, or smoking. The patients included in the study underwent a 2 h Oral Glucose Tolerance Test (OGTT) based on the American Diabetes Association (ADA) criteria (2016) for diagnosis of GDM. Similarly, twenty healthy pregnant women were recruited who had a normal glucose tolerance after OGTT testing (NGT, n = 20). They were followed up till 12 weeks postpartum (FNGT, n = 20), and blood samples were collected. Body mass index was calculated using the standard formula, and systolic and diastolic blood pressure were also recorded (SBP, DBP) from all the subjects.

### 4.2. Clinical Specimen Collection and Biochemical Parameters

Blood samples were collected in sodium fluoride tubes from all recruited subjects (at baseline and follow-up) after overnight fasting and after 1 h and 2 h of ingestion of 75g of glucose. The plasma samples separated from this were used for glucose estimation and biochemical tests. The biochemical tests performed include triglycerides (TGs), total cholesterol, low-density lipoprotein cholesterol (LDL-c), and high-density lipoprotein cholesterol (HDL-c) in an ISO 15189:2012 accredited clinical lab. Plasma samples collected at fasting were also used for miRNA extraction using the miRNeasy Serum/Plasma Kit (Catalog #217184, Qiagen, CA, USA). The extracted miRNAs were then reverse transcribed into a final 10 μL reaction volume comprising 3.75 μL of isolated miRNAs using the Mir-X miRNA First Strand Synthesis Kit (Catalog #638313, Takara Bio USA, Inc., San Jose, CA, USA) according to the manufacturer’s instructions. The cDNA was diluted 1:10 using molecular-grade water and stored at −80 °C until use.

### 4.3. RT-qPCR for miRNA Expression

Three miRNAs were selected for this study based on previous literature, namely, hsa-miR-16-5p, hsa-miR-17-5p, and hsa-miR-20a-5p. For RT-qPCR, 3 μL of 10 times diluted cDNA was used in a 10 μL PCR reaction using GoTaq qPCR Master mix (Catalog #A6001, Promega Corporation, Madison, WI, USA). For the normalization of miRNA expression data, snRNA U6 (Takara Mir-X miRNA First strand synthesis kit) was employed as an endogenous control. This selection was based on its prevalent use in previous miRNA studies [38,39]. We used the comparative ΔCt method to ensure its appropriateness for our study conditions [40]. A Takara Mir-X miRNA first strand synthesis kit supplied the forward and reverse primers for the endogenous control and the universal reverse primer. The forward primers used for each miRNA are as follows: hsa-miR-16-5p: 5′-TAGCAGCAGCACGTAAATATTGGCG-3′; hsa-miR-17-5p: 5′-CAAAGTGCTTACAGTGCAGGTAG-3′; and hsa-miR-20a-5p: 5′-TAAAGTGCTTATAGTGCAGGTAG-3′. The RT-qPCR was performed in a 7500 Real Time PCR System (Applied Biosystems, Foster City, CA, USA). The cycling conditions used were according to the GoTaq qPCR Master Mix catalogue manual. Each sample was run in duplicate. At the end of the PCR, melting curve analysis was performed as a quality check for amplification specificity. The software SDS v.1.5.1 was used to evaluate the amplification, Ct-value, and melting curve analysis (Applied Biosystems, MA, USA). The relative expression of miRNA was calculated using the 2^−ΔCT^ method.

### 4.4. Statistical Analysis

The statistical analysis was performed using the ‘Rcmdr’ package of the R software (R version 4.2.0). The normality of the data obtained was analyzed using the Shapiro–Wilk test. The data were presented as mean ± standard deviation (SD) or median and interquartle range (IQR). A paired *t*-test was used for data showing normal distribution, and a two-sample Wilcoxon test was used for data that did not show normal distribution. Correlation analysis was performed using the Spearman test. The diagnostic utility of each miRNA was assessed with receiver operating characteristic (ROC) curve analysis in R using the ‘pROC’ package.

## 5. Conclusions

In conclusion, this longitudinal validation study has illuminated the expression trajectories of hsa-miR-16-5p, hsa-miR-17-5p, and hsa-miR-20a-5p in GDM and postpartum. The past studies have analyzed the miRNAs involved in GDM at a certain point in pregnancy. This study has analyzed the miRNA expression over the gestational period and postpartum to try to derive a link between GDM reversal, postpartum, and miRNA expression. While hsa-miR-16-5p shows strong promise as a diagnostic biomarker for early detection of GDM, hsa-miR-17-5p and hsa-miR-20a-5p are valuable as exploratory signals that require further validation in larger cohorts for their diagnostic utility. The persistent elevation of hsa-miR-16-5p and hsa-miR-17-5p postpartum strongly suggests their role in GDM progression to type 2 diabetes mellitus. These findings underscore the pivotal role of these microRNAs in GDM and its postpartum trajectory, particularly in contributing to the elevated risk of type 2 diabetes mellitus among previously affected mothers. This knowledge could prove essential in the early detection and management of insulin resistance during pregnancy and postpartum. Future comparative studies exploring other epigenetic factors and changes at the tissue level are warranted to further improve these findings.

## Figures and Tables

**Table 1:** Clinical characteristics and miRNA expression levels in NGT vs. GDM.

Variables	NGT (n= 20)	GDM (n = 30)	*p* Value
Age (mean ± SD)	26.30 ± 3.88	26.83 ± 4.62	0.662
BMI	22.30 (4.53)	28.65 (6.76)	0.004
DBP	100 (25)	112 (16)	0.552
SBP (mean ± SD)	58.85 ± 10.23	65.57 ± 9.85	0.026
TGs (mg/dL)	153.09 (47.04)	203.20 (95.72)	0.0004
Total cholesterol (mg/dL) (mean ± SD)	199.07 ± 46.88	181.52 ± 46.34	0.200
LDL (mg/dL) (mean ± SD)	123.24 ± 42.29	98.64 ± 41.30	0.048
HDL (mg/dL) (mean ± SD)	43.78 ± 10.10	39.11 ± 12.02	0.145
FBG (mg/dL)	81.5 (8.50)	94.5 (4.75)	0.0000005
1 hr BG (mg/dL) (mean ± SD)	109.95 ± 23.84	151.67 ± 37.05	0.00001
2 hr BG(mg/dL)	86 (20.00)	128 (73.25)	0.00001
hsa-miR-16 (2^−dCq^)	22.52 (24.42)	106.44 (115.21)	0.004
hsa-miR-17 (2^−dCq^)	3.35 (8.94)	10.18 (15.30)	0.044
Has-miR-20a (2^−dCq^)	4.51 (5.29)	10.56 (20.76)	0.044

NGT: normal glucose tolerance, GDM: gestational diabetes mellitus, n: number of subjects, SD: standard deviation, BMI: body mass index, DBP: diastolic blood pressure, SBP: systolic blood pressure, TGs: triglycerides, LDL: low-density lipoprotein, HDL: high-density lipoprotein, FBG: fasting blood glucose, 1 hr BG: 1 h post meal blood glucose, 2 hr BG: 2 h post meal blood glucose, Cq: cycle of quantification.

**Table 2:** Clinical characteristics and miRNA expression levels in GDM vs. FGDM.

Variables	GDM (n= 30)	FGDM (n = 30)	*p* Value
TGs (mg/dL)	203.2 (95.72)	191.31 (117.50)	0.0196
Total cholesterol (mg/dL) (mean ± SD)	181.52 ± 46.34	184.57 ± 37.45	0.807
LDL (mg/dL) (mean ± SD)	90.65 (32.98)	100.20 (27.83)	0.515
aHDL (mg/dL) (mean ± SD)	39.11 ± 12.02	47.39 ± 11.98	0.0254
hsa-miR-16-5p (2^−dCq^)	106.44 (115.21)	110.06 (314.94)	0.100
hsa-miR-17-5p (2^−dCq^)	10.17 (15.29)	19.97 (31.83)	0.026
hsa-miR-20a-5p (2^−dCq^)	10.56 (20.76)	17.03 (42.44)	0.134

**Table 3:** Clinical characteristics and miRNA expression levels in FNGT vs. FGDM.

Variables	FNGT (n = 20)	FGDM (n = 30)	*p* Value
TG (mg/dL)	92.21 (125.04)	191.32 (117.49)	0.116
Total cholesterol (mg/dL) (mean ± SD)	162.84 ± 37.48	184.57 ± 37.45	0.051
LDL (mg/dL) (mean ± SD)	94.58 ± 30.28	102.49 ± 23.90	0.332
HDL (mg/dL) (mean ± SD)	42.19 ± 11.71	47.39 ± 11.98	0.134
RBS postpartum	80.95 (8.49)	95.54 (17.13)	0.00078
hsa-miR-16-5p (2^−dCq^)	34.86 (42.92)	110.06 (314.94)	0.005
hsa-miR-17-5p (2^−dCq^)	18.29 (42.56)	19.97 (31.83)	0.428
hsa-miR-20a-5p (2^−dCq^)	16.76 (29.40)	17.03 (42.44)	0.301

**Table 4:** Clinical characteristics and miRNA expression levels in NGT vs. FNGT.

Variables	NGT (n = 20)	FNGT (n = 20)	*p* Value
TG (mg/dL)	153.09 (47.04)	92.21 (125.04)	0.2774
Total cholesterol (mg/dL) (mean ± SD)	199.07 ± 46.88	162.84 ± 37.48	0.01718
LDL (mg/dL) (mean ± SD)	123.24 ± 42.29	94.58 ± 30.28	0.03459
aHDL (mg/dL) (mean ± SD)	43.78 ± 10.10	42.19 ± 11.71	0.6541
hsa-miR-16-5p (2^−dCq^)	22.52 (24.42)	34.86 (42.92)	0.7012
hsa-miR-17-5p (2^−dCq^)	3.35 (8.94)	18.29 (42.56)	0.1893
hsa-miR-20a-5p (2^−dCq^)	4.51 (5.29)	16.76 (29.40)	0.2162

## Data Availability

The raw data supporting the conclusions of this article will be made available by the authors upon request.

## Supplementary Materials

The following supporting information can be downloaded at: https://www.mdpi.com/article/10.3390/epigenomes9040037/s1, Table S1. Correlation analysis of miRNA and other variables in baseline samples of NGT and GDM.

## Abbreviations

ADA—American Diabetes Association; BMI—Body Mass Index; cDNA—complementary DNA; Ct Value—Cycle Threshold Value; FGDM—Postpartum Follow-Up Samples of GDM Individuals; FNGT—Postpartum Follow-Up Samples of NGT Individuals; GDM—Gestational Diabetes Mellitus; HDL-c—High-Density Lipoprotein Cholesterol; HOMA-IR—Homeostatic Model Assessment of Insulin Resistance; IEC No—Institutional Ethics Committee Number; ISO—International Organization for Standardization; LDL-c—Low-Density Lipoprotein Cholesterol; miRNA—micro RNA; NGT—Normal Glucose Tolerance; OGTT—Oral Glucose Tolerance Test; OPD—Out-Patient Department; PCR—Polymerase Chain Reaction; RNA—Ribonucleic acid; ROC—Receiver Operator Characteristic; RT-qPCR—Quantitative Reverse Transcription PCR; SBP—Systolic Blood Pressure; T2DM—Type 2 Diabetes Mellitus; TGs—Triglycerides.
