# Supplementary material for: Sustained Higher Levels of Plasma hsa-miR-17-5p Expression During Gestational Diabetes Mellitus and Postpartum
Source: Epigenomes. 2025 Sep 24;9(4):37. doi: 10.3390/epigenomes9040037 (PMC12551101; doi:10.3390/epigenomes9040037)
Supplement: Supplementary file 1 [file epigenomes-09-00037-s001.zip › epigenomes-3831247-supplementary.pdf]

Table S1. Correlation analysis of miRNA and other variables in baseline samples of NGT and GDM:

	**miR 16**		**miR 17**		**miR20a**	
	**rho value**	**P value**	**rho value**	**P value**	**rho value**	**P value**
Age (mean ± SD)	-0.055	0.704	0.035	0.808	-0.031	0.829
BMI	0.107	0.460	0.256	0.164	0.109	0.450
DBP	-0.009	0.947	0.034	0.814	0.003	0.983
SBP (mean ± SD)	0.272	0.056	0.262	0.066	0.274	0.054
TG (mg/dL)	0.134	0.215	0.226	0.115	0.201	0.162
Total Cholesterol (mg/dL) (mean ± SD)	-0.086	0.554	-0.099	0.495	-0.045	0.754
LDL (mg/dL) (mean ± SD)	-0.079	0.585	-0.078	0.588	-0.032	0.827
aHDL (mg/dL) (mean ± SD)	0.152	0.292	0.081	0.577	0.143	0.321
FBG (mg/dL)	0.242	0.089	0.061	0.672	0.104	0.474
1hr BG (mg/dL) (mean ± SD)	0.230	0.108	0.223	0.119	0.189	0.189
2hr BG(mg/dL)	0.106	0.466	0.128	0.374	0.094	0.517
hsa-miR-16 (2^{-dCq})			0.827	$1.32 \times e^{-13}$	0.894	$< 2.2 \times e^{-16}$
hsa-miR-17 (2^{-dCq})	0.827	$1.32 \times e^{-13}$			0.947	$< 2.2 \times e^{-16}$
Has-miR-20a (2^{-dCq})	0.894	$< 2.2 \times e^{-16}$	0.947	$< 2.2 \times e^{-16}$		
